# High HIV Detection in a Tertiary Facility in Liberia: Implications and Opportunities

**DOI:** 10.5334/aogh.3243

**Published:** 2021-11-25

**Authors:** Onyema Ogbuagu, Ian Wachekwa, Faiza Yasin, Cecilia Nuta, Sean Donato, Julia Toomey, Mukhtar Adeiza, Lydia Aoun Barakat

**Affiliations:** 1 Section of Infectious Diseases, Yale University School of Medicine, Connecticut, New Haven, USA; 2 Department of Internal Medicine, John F. Kennedy Medical Center, Monrovia, LR; 3 Office of Global Health, Department of Medicine, Yale University School of Medicine, Connecticut, New Haven, USA; 4 One Medical, San Francisco, USA; 5 Department of Pediatrics, John F. Kennedy Medical Center, Monrovia, LR; 6 UNICEF supply division, Copenhagen, DK; 7 National AIDS Control Program, LR

## Abstract

**Background:**

HIV/AIDS remains one of the world’s most significant public health challenges; sub-Saharan Africa accounts for 71% of the global burden of HIV. Testing for HIV is pivotal to achieving UNAIDS 95-95-95 target towards bringing an end to the epidemic.

**Objective:**

The study assessed five-year HIV testing data from the largest tertiary hospital in Monrovia, Liberia and highlights risk groups that would benefit from targeted testing and prevention interventions.

**Methods:**

This was a single-center academic hospital-based retrospective analysis of HIV testing data from January 2014 to December 2018 obtained from all testing sites at John F. Kennedy Medical Center in Monrovia, Liberia. Pooled HIV testing data during the study period were analyzed using descriptive statistics and stratified by age, gender and pregnancy status. Annual diagnoses rates were reported as proportion of individuals tested within a specified category (age [<15 years, age 15–24 years and >=25 years], gender, and pregnancy status) that had a positive HIV test. Five-year trends were analyzed.

**Results:**

Over the study period, 41,343 non-pregnant individuals were screened for HIV. In addition, the antenatal clinic performed 24,913 tests. Of non-pregnant individuals tested, 4,066 (10%) were diagnosed with HIV ranging from 7% (909/12821) in 2018 to 13% (678/5079) in 2014. Case detection rates for individuals aged 15–24 were 7%, 5%, 4%, 6% and 3% for years 2014, 2015, 2016, 2017 and 2018 respectively. Annually, 2–3% of all pregnant women tested were diagnosed with HIV. While HIV detection rates decreased over time overall, children less than 15 years of age showed an annual increase from 6.7% in 2014 to 12.3% in 2018.

**Conclusion:**

A large five-year dataset from the largest tertiary facility in Liberia shows broad HIV detection rates that are much higher than national prevalence estimates. Ramping up HIV testing and prevention interventions including pre-exposure prophylaxis are sorely needed.

## Background

HIV infection is a leading cause of mortality in low and middle-income countries (LMICs). Sub-Saharan Africa (SSA), home to a disproportionate number of people living with HIV (PLWH), accounts for about 68% of the global burden [1]. Within the subcontinent, there is wide variation in HIV prevalence with West and Central Africa regions having an estimated prevalence of 1.5% [2]. West Africa, in particular, faces tremendous challenges with its HIV/AIDS response including low rates of HIV testing such that an estimated 35% of PLWH remain unidentified and suboptimal combination antiretroviral therapy (cART) coverage rates all exacerbated by HIV-related stigma, poverty and other structural barriers to accessing healthcare services [3].

Liberia, in West Africa, has faced its own unique challenges including that its basic healthcare infrastructure was destroyed by 14 years of civil wars and, more recently, the 2014–2016 Ebola outbreak that decimated its healthcare workforce [4]. In spite of these, Liberia’s reported HIV prevalence decreased significantly, declining from 2.7–12.4% in 2004 [5] to 1.3% in 2018 [2]. The decline was attributed to the formation of the National AIDS Control Program that supported training health care providers on and establishment of sites for voluntary counseling and testing (VCT), implementation of programs to prevent maternal to child transmission (PMTCT), condom promotion and expanded utilization of cART [2,3,5]. However, the declining proportions could be also related to poor testing rates leading to underestimation of the disease prevalence.

Within Liberia, HIV prevalence has been observed to be unevenly distributed being higher among certain subgroups including urban area dwellers (2.6% in capital city of Monrovia compared to 0.8% in rural areas), female sex workers (FSWs) (16.7%), men who have sex with men (MSM) (37.9%), transgender (TG) (27.6%), and persons who inject drugs (PWID) (14.4%) [2018/2019 integrated bio-behavioral surveillance survey]. However, HIV testing among the general population is significantly lacking. This was documented in the 2019–2020 Liberia Demographic and Health Survey (DHS) [6], where among respondents 15–49 years of age, 66% of men and 45% of women had never been tested for HIV [2]. Therefore, data from Liberia are incomplete, and highlights that current testing strategies are insufficient to meet the 2030 UNAIDS 95-95-95 target.

Here, we describe HIV testing data obtained over a five-year period from a voluntary counseling and testing programs within the largest tertiary care hospital in Monrovia.

## Methods

This retrospective study analyzed data obtained from John F. Kennedy Medical Center (JFKMC) in Monrovia, Liberia, the largest hospital and tertiary care center (400 bed capacity) in Liberia from January 2014 to December 2018. It provides health care services to and receives referrals from all 15 counties of Liberia (population – 4.9 million.) At the location, HIV testing, and counseling (HTC) is conducted by dedicated teams of lay/peer or trained counselors, and/or clinicians located at the adult and pediatric HIV clinics, antenatal clinic (ANC), medical and surgical wards, outpatient departments and emergency room. HIV testing is conducted voluntarily when sought by patients while others are referred to the program by their medical providers. HIV-1/2 testing is conducted utilizing rapid tests (Determine®, Alere Medical Co Ltd., Matsudo-shi, Japan; Bioline® Standard Diagnostic Inc, Kyonggi-do, Republic of Korea; or Unigold® Trinity Biotech PLC, Co Wicklow, Ireland.) Positive tests are confirmed with another rapid test. Discordant tests are repeated with confirmatory samples sent to the main laboratory for ELISA testing. Patients with positive tests are referred to the HIV clinic.

Data collected from testing program attendees included age, gender, and pregnancy status. Testing data from the facility are aggregated by month and year, deidentified and fed into the national Health Management Information System (HMIS) within the Ministry of Health.

Pooled HIV testing data from all testing sites at the facility during the study period were analyzed and reported using descriptive statistics and stratified by pregnancy status (data on males and non-pregnant females were pooled while data from pregnant women were reported separately). Annual diagnoses rates were reported as proportion of individuals tested within a specified category (age [<15 years, age 15–24 and >=25 years], gender, and pregnancy status) that had a positive HIV test.

## Patient Consent Statement

Patient consent was not required as the data used in this analysis are collected as part of routine deidentified HIV surveillance and reporting. As it was deemed non-research, permission to use the data was obtained from the Liberian John F. Kennedy Hospital HIV program and Hospital Administration.

## Results

Over the five-year study period, 41,343 tests were performed among non-pregnant individuals. The antenatal clinic performed 24,913 tests on pregnant women from 2014–2018. HIV testing volume was relatively lower in 2014 and 2015 coincident with the Ebola Virus Disease (EBV) epidemic in the country [see ***Table 1***.] Overall, 2184 and 7,875 tests were conducted among children aged <15 years, and older teenagers and young adults (comprised of males and non-pregnant females) between ages 15–24 years respectively.

**Table 1:** Proportion of positive HIV tests by gender, age and pregnancy status.


YEAR	CATEGORY	NUMBER TESTED	NUMBER POSITIVE	PERCENT POSITIVE

2014	Total (non-pregnant)	5079	678	13.3%

	Males	2175	204	9.4%

	Females	2904	474	16.3%

	Age <15 yrs	702	47	14.9%

	Age 15–24	742	55	7.4%

	Pregnant Women	3905	111	2.8%

2015	Total (non-pregnant)	5126	636	12.4%

	Males	2430	224	9.2%

	Females	2696	412	15.3%

	Age < 15 yrs	336	34	10.1%

	Age 15–24	876	41	4.7%

	Pregnant Women	4077	98	2.4%

2016	Total (non-pregnant)	10304	1057	10.3%

	Males	4848	372	7.7%

	Females	5456	685	12.6%

	Age <15 yrs	392	43	11.0%

	Age 15–24	2605	95	3.6%

	Pregnant Women	5061	120	2.4%

2017	Total (non-pregnant)	8013	786	9.8%

	Males	4031	270	6.7%

	Females	3982	516	13.0%

	Age < 15 yrs	388	45	11.6%

	Age 15–24	1170	69	5.9%

	Pregnant Women	5106	114	2.2%

2018	Total (non-pregnant)	12821	909	7.1%

	Males	6268	339	5.4%

	Females	6553	570	8.7%

	Age < 15 yrs	366	45	12.3%

	Age 15–24	2482	83	3.3%

	Pregnant Women	6764	102	1.5%


**Figure 1:**
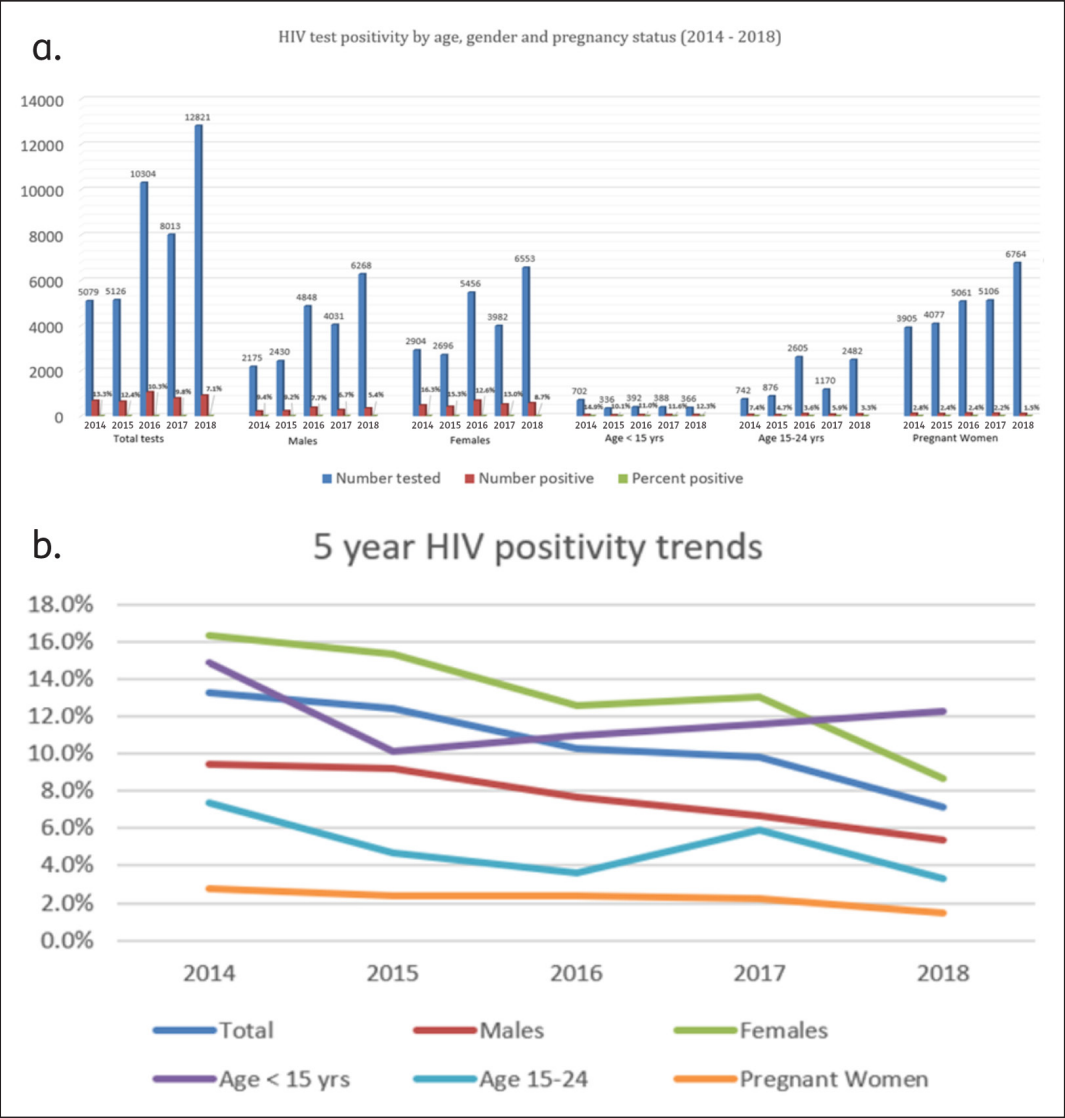
**a**. HIV test positivity by age, gender and pregnancy status (2014–2018). **b**. 5-year HIV positivity trends.

Overall, 4,066 males and non-pregnant females were diagnosed with HIV, a case detection rate of 9.8%. For that group, HIV diagnoses ranged from 13.3% (678/5079) in 2014 to 7.1% (909/1282) in 2018 with a steady decline observed over the five-year period [see ***Figure 1 a*** and ***b***.] Non-pregnant women had the highest case detection rates ranging from 16.3% (2014) to 8.7% (2018) while for males it was 9.4% in 2014 and 5.4% in 2018. HIV detection rates declined over the five-year study period for both groups. Children less than 15 years of age showed an annual increase from 6.7% in 2014 to 12.3% in 2018. For individuals aged 15–24 years, positive tests occurred among 7.4%, 4.7%, 3.6%, 5.9% and 3.3% for years 2014, 2015, 2016, 2017, and 2018 respectively. From 2014–2018, 1.5–2.8% of all pregnant women tested positive for HIV infection.

## Discussion

This five-year data from the largest hospital in Liberia showed HIV detection rates that are well above the national reproductive age prevalence estimates of 1.5% (UNAIDS, 2019). Specifically, the data showed that the non-pregnant women and reproductive age men tested yielded high proportions of positive tests though detection rates showed declines over time. The declining test positivity suggests a pool of infected but previously unidentified individuals that are being detected and thereby, depleted. With only 58% of PLWH in Liberia being aware of their infection, this highlights the need to ramp up testing efforts. Otherwise, children less than 15 years of age also demonstrated high rates of HIV detection and the rise in cases detected over the five-year period likely represents identification of previously undetected perinatally acquired infections. This is buttressed by the volume of HIV infections detected in ANC programs and high rate of perinatal transmission in Liberia (15%).

Women in Liberia face unique challenges that put them at risk for HIV. They report very young age at sexual debut, multiple partners, high teen pregnancy rates (55%), and low rates of condom use [6], thus prevention services are a critical need. Furthermore, individuals aged 15–24 years, regardless of gender, typically have lower levels of knowledge about HIV in general including risk behaviors and prevention modalities.

The high rates of infections among men were likely driven by sampling, as men who seek testing are more likely to be at high risk for the infection or do so when symptomatic. The disparity in HIV testing rates between men and women has been well documented in Sub-Saharan Africa (SSA) [7] as was identified in Liberia’s 2019–2020 DHS survey that showed that while men and women were equally aware of where HIV testing was performed, 50% of women and only 32% of men had ever been tested for HIV [6].

The first step in the HIV treatment cascade is HIV testing without which meeting UNAIDS 95-95-95 targets would be impossible. In Liberia, suboptimal laboratory capacity and testing kit supply chain problems are obstacles to ramping up HIV testing. Even when these barriers are successfully addressed, innovative and intensive HTC strategies are warranted to engage individuals in the community setting including strategies utilizing community-based and rapid point-of-care testing (POCT) that have proven effective in LMICs, and with at-risk populations [8,9,10,11]. Peer-driven HIV testing has also been associated with increased acceptance [10]. HIV self-testing (HIVST) can also play a substantial role in improving testing [9].

Fortunately, Liberia is now rebuilding its healthcare infrastructure to position itself better to address its public health challenges. Implementing robust testing for individuals at risk of acquiring HIV allows for early engagement in care, but also decreases the chance that such individuals would transmit the virus to others. Other evidence-based prevention services such as pre-exposure prophylaxis (PrEP) should be scaled up for non-infected high-risk individuals similar to what has been done with antiretroviral treatment programs, with the potential to greatly impact HIV prevalence [12].

Our study has some limitations. Individuals seeking HIV testing may represent those at increased risk thereby resulting in increased case detection rates. As testing was performed primarily in pregnant women and urban city dwellers, the study findings may not be generalizable to cohorts with dissimilar characteristics. We did not assess specific HIV risk behaviors that would have enabled us to better evaluate if individuals in the study were at relatively higher or lower risk of acquiring HIV compared to the general population.

In conclusion, a large five-year dataset from the largest tertiary facility in Liberia shows HIV detection rates that are much higher than national prevalence estimates. Ramping up HIV testing and prevention interventions including pre-exposure prophylaxis are sorely needed.

## Disclaimer

The contents are those of the authors and do not necessarily represent the official views of, nor an endorsement, by HRSA, HHS or the U.S. government.

## References

[1] Avert. HIV and AIDS in West and Central Africa Overview, 2019. Available at: https://www.avert.org/hiv-and-aids-west-and-central-africa-overview. Accessed 2 November 2020.

[2] Monitoring and Evaluation Department of the National AIDS and STI Control Program. Liberia HIV&AIDS Response Progress Report, 2016. Available at: https://www.unaids.org/sites/default/files/country/documents/LBR_narrative_report_2016.pdf. Accessed 2 November 2020.

[3] US President’s Emergency Plan for AIDS Relief (PEPFAR). West Africa Regional Program Regional Operational Plan 2019 Strategic Direction Summary. Available at: https://www.state.gov/wp-content/uploads/2019/09/West-Africa_ROP19-Strategic-Directional-Summary_public-FINAL.pdf. Accessed 2 November 2020.

[4] Tattevin P, Baysah MK, Raguin G, et al. Retention in care for HIV-infected patients in the eye of the Ebola storm: lessons from Monrovia, Liberia. AIDS (London, England). 2015; 29(6): N1–N2. DOI: 10.1097/QAD.0000000000000614 25849843

[5] United Nations Development Programme. United Nations Development Programme Partnership with the Global Fund in Liberia: Supporting implementation, developing capacity. 2010. Available at: https://www.undp.org/content/dam/aplaws/publication/en/publications/capacity-development/case-study-on-undps-partnership-with-the-global-fund-in-liberia/100WATT-UNDP-liberiaBD.pdf. Accessed 2 November 2020.

[6] Liberia Institute of Statistics and Geo-Information Services (LISGIS), Ministry of Health and Social Welfare [Liberia], and ICF. 2020. Liberia Demographic and Health Survey 2019–20: Key Indicators. Available at: https://dhsprogram.com/pubs/pdf/PR117/PR117.pdf. Accessed 2 November 2020.

[7] Carrasco MA, Fleming P, Wagman J, Wong V. Toward 90-90-90: Identifying those who have never been tested for HIV and differences by sex in Lesotho. AIDS care. 2018; 30(3): 284–8. DOI: 10.1080/09540121.2017.1372559 28868903

[8] Lazarus L, Patel S, Shaw A, et al. Uptake of Community-Based Peer Administered HIV Point-of-Care Testing: Findings from the PROUD Study. PloS one. 2016; 11(12): e0166942–e. DOI: 10.1371/journal.pone.0166942 27911908 PMC5135055

[9] Chanda MM, Ortblad KF, Mwale M, et al. HIV self-testing among female sex workers in Zambia: A cluster randomized controlled trial. PLoS med. 2017; 14(11): e1002442–e. DOI: 10.1371/journal.pmed.1002442 29161260 PMC5697803

[10] Veronese V, Oo ZM, Thein ZW, et al. Acceptability of Peer-Delivered HIV Testing and Counselling Among Men Who Have Sex with Men (MSM) and Transgender Women (TW) in Myanmar. AIDS behav. 2018; 22(8): 2426–34. DOI: 10.1007/s10461-017-2022-0 29427231

[11] Yan H, Zhang R, Wei C, et al. A peer-led, community-based rapid HIV testing intervention among untested men who have sex with men in China: An operational model for expansion of HIV testing and linkage to care. Sex transm infect. 2014; 90(5): 388–93. DOI: 10.1136/sextrans-2013-051397 24926040

[12] Cowan FM, Delany-Moretlwe S, Sanders EJ, et al. PrEP implementation research in Africa: what is new? J Int AIDS Soc. 2016; 19(7(Suppl 6)): 21101. DOI: 10.7448/IAS.19.7.21101 27760680 PMC5071780

